# Supplementary material for: CD36 identified by weighted gene co-expression network analysis as a hub candidate gene in lupus nephritis
Source: PeerJ. 2019 Oct 1;7:e7722. doi: 10.7717/peerj.7722 (PMC6777479; doi:10.7717/peerj.7722)
Supplement: Document S1 [file peerj-07-7722-s004.docx]

**The dataset of *Peterson Lupus Glom***

**Description:**

25 laser captured lupus glomeruli from 12 different biopsies together with 6 control glomeruli from 4 biopsies were analyzed on Corning GAPS slides (Corning Life Sciences, Acton, Massachusetts, USA) using an Amersham Biosciences Generation III spotter (Amersham Biosciences, Piscataway, New Jersey, USA). The B12 array contained 3,602 genes and the B13 array had the same 3,602 genes plus an additional 428 genes. Each clone was spotted in duplicate on the array. This dataset was previously named Peterson Lupus.

**Related Study:**

Characterization of heterogeneity in the molecular pathogenesis of lupus nephritis from transcriptional profiles of laser-captured glomeruli. [PubMed]

**Datalink(s):**
<http://www.jci.org/cgi/content/full/113/12/1722/DC1>

**The dataset of *Berthier Lupus Glom***

**Description:**

Expression data from human with lupus nephritis (LN). LN is one of the most severe complications of the rheumatologic autoimmune disease called systemic lupus erythematosus (SLE). We used microarrays to analyze the transcriptome of microdissected renal biopsies from 32 patients with LN compared to 15 healthy pre-transplant living donor controls. RNA from glomerular compartments was extracted and processed for hybridization on Affymetrix HG-U133A microarrays. RNA from corresponding tubulointerstitial tissue can be found in the Berthier Lupus TubInt dataset. These samples were previously available as part of the Berthier Lupus dataset, which combined both glomerular and tubulointerstitial samples. Note: these samples are part of the ERCB cohort described fully in the Ju CKD Glom and Ju CKD TubInt datasets.

**Study:**

Cross-species transcriptional network analysis defines shared inflammatory responses in murine and human lupus nephritis. [[PubMed](http://www.ncbi.nlm.nih.gov/pubmed/22723521?dopt=Abstract)]

**Datalink(s):**
<http://www.ncbi.nlm.nih.gov/geo/query/acc.cgi?acc=GSE32591>
